# Supplementary material for: Derivation and validation of a model to predict treatment failure among under five children with severe community acquired pneumonia who are admitted at Debre Tabor specialized comprehensive hospital
Source: PLoS One. 2025 Mar 26;20(3):e0320448. doi: 10.1371/journal.pone.0320448 (PMC11940812; doi:10.1371/journal.pone.0320448)
Supplement: Attachment — Submitted filename: Point-by-point response to reviewers.pdf [file pone.0320448.s006.pdf]

Derivation and validation of a model to predict treatment failure among under five children with severe community acquired pneumonia who are admitted at Debre Tabor specialized comprehensive hospital

Muluken Chanie Agimas*[1], Tigabu Kidie Tesfie[1], Nebiyu Mekonnen Derseh[1], Amare Kassaw[2]

[1] Department of Epidemiology and Biostatistics, institute of public health, college of medicine and health science, University of Gondar, Gondar, Ethiopia.

[2] Department of pediatrics and child health Nursing, College of Health sciences,

Debre Tabor University, Debre Tabor, Ethiopia

Authors address:

1. Muluken Chanie Agimas (MCA): mulukensrc12@gmail.com
2. Tigabu Kidie Tesfie (TKT): tigabukidie@gmail.com
3. Nebiyu Mekonen Derseh (NMD): nebiyumek12@gmail.com
4. Amare Kassaw(AK): amarekassaw2009@gmail.com

Corresponding author:

Muluken Chanie Agimas (MCA): mulukensrc12@gmail.com

To: PLOS ONE

Submission ID: PONE-D-23-36484

Cover Letter for reviewer’s and editor comments and authors responses

We would like to thank both the Editorial board members and reviewers for kind comments and suggestions. Accordingly, we have tried to correct and respond to those comments point by point, page by page, and line by line. Therefore, we prepared this author’s response, the revised manuscript and the tracked changes of the manuscript. We accept most of your comments and some of your comments are needs justification to be clear for the scientific community.

Sincerely, with best regards!

Muluken Chanie Agimas

ID: https://orcid.org/0000-0003-0808-124X

**Comments from review and editorial board**	**Authors response**
**Editors comment**	**Authors response**
Please ensure that your manuscript meets PLOS ONE's style requirements, including those for file naming.	Thank you dear editors for you suggestion. I admit the comment and correction has been made accordingly.
Please provide a complete Data Availability Statement in the submission form, ensuring you include all necessary access information or a reason for why you are unable to make your data freely accessible. If your research concerns only data provided within your	Thank you dear editors for you suggestion. I admit the comment and correction has been made accordingly.

submission, please write "All data are in the manuscript and/or supporting information files" as your Data Availability Statement.	
Your ethics statement should only appear in the Methods section of your manuscript. If your ethics statement is written in any section besides the Methods, please delete it from any other section.	Thank you dear editors for you suggestion. I admit the comment and correction has been made accordingly.
Please include your tables as part of your main manuscript and remove the individual files. Please note that supplementary tables should be uploaded as separate "supporting information" files.	Thank you dear editors for you suggestion. I admit the comment and correction has been made accordingly.
Please include captions for your Supporting Information files at the end of your manuscript, and update any in-text citations to match accordingly.	Thank you dear editors for you suggestion. I admit the comment and correction has been made accordingly.
Reviewer one comments	Authors response
The independent variable better rearrange in theme like socio-demographic, health facility related factors, Individual related factors, …backward step-wise logistic regression analysis also follow this theme.	Thank you so much for your recommendation. Accordingly the necessary amendment has made. Dear reviewer we didn’t use health facility related factors. Because we used already collected data or secondary data. Thus based on your recommendation we try to theme the variable

	based on socio-demographic characteristics and baseline disorder/status.
Reviewer two comment	Authors response
Abstract:<br>Check the grammar and write the objective of the research separately	Thank you dear reviewer we have made the necessary amendment.
In the abstract, write the figures in to two digits	Thank you dear reviewer your suggestion but the reason why we used the digit is to report the most precise report. Otherwise the model may have similar AUC finding. To detect this difference, we used the actual digits.
Introduction:<br>In this section, the authors should address the following points<br>What is the clinical importance of the research?	Dear reviewer, the clinical importance of the finding had good discriminatory performance and clinical importance for the treatment of severe community-acquired pneumonia. By early classification and detection of the treatment failure, the finding can reverse the death rate of many children associated with treatment failure.
What is the gap of the previous research?	Majority of the study focused on factors for SCAP. Little is known about the prediction model of treatment failure.
Methods:<br>Use the appropriate punctuation for line 123	Thank you accepted and corrected.
In line 159 please specify the figure.	Thank you accepted and corrected.

What is the importance of accuracy by chance	
Why you used a systematic random sampling?	Dear reviewer actually this was used by the previous researcher. Because we used the secondary data source so just we report what the previous data collector used. Any ways the reason why they used systematic random sampling is because of no frame available to use simple random sampling method.
Results:<br>Please include the table within the main manuscript	Accepted and revised accordingly.
The model "P (SCAP treatment failure) = 1/e-(-2.67 +1.14 *HIV status yes +0.765* plural effusion (yes) +0.55193 *anemia (yes) +0.647*measles (yes) +0.915*chest indrowing (yes) +1.07 *history of SCAP (yes) + 194 0.603*pulse rate (abnormal) (Table-3)" is not well written please revise it.	Thank you dear reviewer. Amendment has made accordingly.

Why you report decision curve analysis?	We used the decision curve analysis to assess the standardized net benefit of the predictive model.
Discussion:<br>If similar studies are available so far, please include additional comparisons.	Dear reviewer we try to search via so many searching engine and we found what we have cited in our discussion part. Lack of sufficient literature makes hard to compare with previous findings.
In line 228, what does it mean 450?	Sorry for the editorial error, instead it mean 45^0
What is the strength of the study?	Using easily measurable predictors in the routine health care system that suits Ethiopia was the strength of the study.
How to compare the AUC with corresponding previous AUC findings?	Nice question. We can compare the AUC using the confidence interval of the current study. If the previous study AUC within the current AUC confidence interval, it is in line with our study finding.
What is your recommendations for researcher in the future?	We recommend that the future researcher focus on large scale study by increasing the sample size and prospective cohort study.

Abstract:<br><br>One of the most important aims of prognostic study is to find out prognostic determinants of the outcome of interest, however, your conclusion of your study in the abstract lacks this important facets of a conclusion.	Thank you for your valuable comment. We accept the comment and correction has made accordingly.
Reviewer three comment	Authors response
The authors should have exclusively searched whether there was a study conducted so far on SCAP in the Ethiopia. The authors deny there is no single study done on the topic, however, there was one study here I dropped the link: https://pubmed.ncbi.nlm.nih.gov/36791115/. The study predicted poor outcomes of SCAP; treatment failure (prognostic outcome in the current study) was one of the composite outcomes in that study. Therefore, the authors in the current study should state and should properly justify that the current study is unique from the previous study; however, this is a flaw	Dear review, thank you for your comment. The previous author measures the outcome variable of poor outcomes of severe community-acquired pneumonia" including treatment failure or antibiotic change or staying longer in the hospital or death occurred during follow-up days. Dear reviewer we have seen the article you mentioned. Your concern was also the concern the authors. We have discussed this issue in group. It was so controversial. Finally, we argued that comparing the current finding with the article you mentioned is not comparable. Because the previous study measures the outcome variable "poor outcome of SCAP"= treatment failure or antibiotic change or staying longer in the hospital or death occurred during follow-up days. But our

	outcome was only treatment failure of SCAP which was measured by persistence of features of severe pneumonia (cough and/or difficult breathing, with or without fever, fast breathing, or a lower chest wall in drawing where their chest moves in or retracts during inhalation) after initiation of antimicrobial therapy or a worsening clinical condition within 48–72 hours of the commencement of the antibiotics" The two measurement are quite different. So comparing the outcome with different measurement is difficult. To compare studies, we believe that outcome must be measured in similar way. Otherwise, our cooperation might be questionable again.
Methods:<br>What by mean: "A secondary analysis of the previous prospective follow-up study was used among children with 92 SCAP who were admitted to the Debre Tabor specialized hospital intensive care unit" ? is this prospective study? What makes prospective if you use previously collected data from the hospital. You need to correct this.	Thank you so much. It was to mean that the study design used by the previous data collector/investigator. Any ways correction has made accordingly.

Results:<br><br>Your original model resulted an accuracy of AUC 77.14%, however, the AUC of the validated model is 77.19%. How this logically acceptable. You do validation for the sake of minimizing over optimistic results. Validation technique is the same as adjustment the same as avoiding crude result for generalization. Logically, original results are over optimistic compared to validated ones, however, yours is controversial. This requires careful correction in case it is editorial problem or else.	Dear reviewer thank you so much. You are right. The error was occurred during reporting the finding from STATA output. Now the correction has made accordingly.
The purpose of conducting prediction studies is to develop an easy to use risk stratification tool at the end of the study. In your case a nomogram tool; this is very important to have it. However, developing risk stratification tool can result in the loss of accuracy because it is simplification technique. This means always there will be a loss of accuracy from the original models. Therefore, researchers expected to develop a nomogram model, which means need to show the accuracy of nomogram model, so	Thank you so much for your comment. Accepted.

that you will be able to compare the results with original ones.	
To construct the nomogram, it is good to have pulse rate and hemoglobin/hematocrit level as they are without classifying as normal vs. abnormal for the purpose of proper use of nomogram tool in the future. Therefore, include these variables in the model as continuous keeping the natural occurrences of the variables for correct interpretation of the tool. In addition, the author should put examples how to interpret the tool for the audience.	Dear reviewer it is an interesting question. Dear reviewer you are right. Considering the variable as continuous variable can be more advantageous. But, the secondary data nature makes hard to use the data as we want. This means we cannot use the data as we need due to the variables like pulse rate and hemoglobin/hematocrit level was reported as a categorical data. We didn’t find the continuous data of such variables. Regarding interpretation of the tool revision has made accordingly.

The authors didn't well discuss their results with study findings and evidences in the country (in Ethiopia) and global context. The conclusion is a single sentence only about accuracy issue of the model. This very bad not make appropriate conclusion about the mainfindings of the study based on the objectives of the study. Therefore, careful attention should be given and a significant change should be made to improve aforementioned parts of the manuscript.	Dear reviewer, in this study we try to focus on only about treatment failure. Thus we develop a predictive model for SCAP associated treatment failure not any additional poor outcome category like death. Previous studies were address the poor outcome of SCAP such as death, prolong stay at hospital, treatment failure and so on. This is quite difficult compare our findings with previous studies with different outcome measurement. We try to compare our findings only studies with comparable outcome measurement (SCAP treatment failure). The limitation of this study was lack of adequate literatures to discuss or compare. Regarding the conclusion: Thank you so much for your important and critical comment. We have made a mistake. But know we try to revised based on the objectives (Model development and internal validation and incidence).

Thank you so much for your very impressive scientific contribution.
