# Supplementary material for: Derivation and validation of a model to predict treatment failure among under five children with severe community acquired pneumonia who are admitted at Debre Tabor specialized comprehensive hospital
Source: PLoS One. 2025 Mar 26;20(3):e0320448. doi: 10.1371/journal.pone.0320448 (PMC11940812; doi:10.1371/journal.pone.0320448)
Supplement: Attachment — Submitted filename: Point-by-point_response_to_reviewers_auresp_2.pdf [file pone.0320448.s007.pdf]

Derivation and validation of a model to predict treatment failure among under five children with severe community acquired pneumonia who are admitted at Debre Tabor specialized comprehensive hospital

Muluken Chanie Agimas*[1], Tigabu Kidie Tesfie[1], Nebiyu Mekonnen Derseh[1], Amare Kassaw[2]

[1] Department of Epidemiology and Biostatistics, institute of public health, college of medicine and health science, University of Gondar, Gondar, Ethiopia.

[2] Department of pediatrics and child health Nursing, College of Health sciences,

Debre Tabor University, Debre Tabor, Ethiopia

Authors address:

1. Muluken Chanie Agimas (MCA): mulukensrc12@gmail.com
2. Tigabu Kidie Tesfie (TKT): tigabukidie@gmail.com
3. Nebiyu Mekonen Derseh (NMD): nebiyumek12@gmail.com
4. Amare Kassaw(AK): amarekassaw2009@gmail.com

Corresponding author:

Muluken Chanie Agimas (MCA): mulukensrc12@gmail.com

To: PLOS ONE

Submission ID: PONE-D-23-36484

Cover Letter for second round reviewer's comment and authors responses

We would like to thank both the Editorial board members and reviewers for kind comments and suggestions. Accordingly, we have tried to correct and respond to those comments point by point, page by page, and line by line. Therefore, we prepared this author's response, the revised manuscript and the tracked changes of the manuscript. We accept most of your comments and some of your comments are needs justification to be clear for the scientific community.

Sincerely, with best regards!

Muluken Chanie Agimas

ID: https://orcid.org/0000-0003-0808-124X

**Comments from reviewer**	**Authors response**
**reviewer comment**	**Authors response**
Abstract<br>The authors, described severe acquired pneumonia is challenging and only mentioned that treatment failure in SCAP is a severe condition, and stated that there is no study conducted to derivate and validate on treatment failure of SCAP. However, didn't mention nothing what by mean treatment failure is about, it is good to state briefly for the readers of this article; even need to justify the need to conduct a prediction study; you	Thank you dear reviewer for you suggestion. I admit the comment and correction has been made accordingly.

need to tell the audience the importance of developing tool such as a nomogram or risk score in the background section.	
Line 34 to 35; says "A multivariable analysis was conducted, and the model performance was evaluated using calibration and area under the curve". You need to mention which multivariable analysis is that specifically that you used??<br>And need to mention that model performance was evaluated using ROC curve with its AUC and calibration curve; why AUC only mentioned there, it is incomplete in your case.	Thank you dear reviewer for you suggestion. Revision has been made accordingly.
You need to mention, the descriptive statistics you used briefly in the method section, you overlooked, but findings were appeared in the result section.	Thank you dear reviewer for you suggestion. I admit the comment and correction has been made accordingly.

The findings of this study showed that AUC for the original model is less than the validated model (line 41 to 42). How this scenario happened, general expectation is the metrics of the validated model is to be less, however, in your case the AUC is more optimized. I urge the authors to look back and rerun your validated model for accuracy. If still the result happened the same, you need to mention in what case it rarely happens and justify.	Dear reviewer, the AUC for the original model was 0.7719 and for the AUC of the validated model was 0.7714. Thus the AUC of the original model was not lower than the validated one. Still the result is nearly similar. This means when the AUC (Area Under the ROC Curve) for the original model (often the model trained on the full training data) is nearly the same as the AUC for the validated model (typically the model evaluated on a separate validation or test dataset), it generally indicates that the model has good generalization. This means that the model performs well not just on the data it was trained on, but also on unseen data, which is critical for ensuring its robustness in real-world applications.<br>Conditions for Similar AUC:<br>1. No over fitting (using regularization, pruning, early stopping).<br>2. Appropriate model complexity (neither too simple nor too complex).<br>3. Proper cross-validation (k-fold or other techniques).<br>4. No data leakage (validation data must remain unseen during training).

	5. Consistent data distributions (training and validation sets must be similar).<br>6. Balanced dataset (for imbalanced classes).<br>7. Proper hyperparameter tuning (using validation sets correctly).<br>8. Use of ensemble methods to improve robustness and reduce overfitting.<br><br>When these conditions are met, the AUC for the original model (trained on the training data) and the validated model (tested on unseen validation data) will remain similar, suggesting that the model has good generalization and can make reliable predictions on new data.
The conclusion part of your abstract is not well written, there is no conclusion made based on the determinants as one of your main objective to derivate a model, you are not only validating a model. In addition, you mentioned in your conclusion that your model had good discriminatory performance, is accuracy measures of a model only discrimination ability? What about	Dear reviewer, as far as our knowledge the conclusion is written based on our objective. Our objectives were just estimate the incidence of treatment failure, develop and validate the prediction model. Thus we tried to summarize based on these objectives.<br>Discrimination power of a prediction model refers to its ability to correctly distinguish between different classes or outcomes. In

calibration ability, even what about other measures of accuracy? Even what by mean good discrimination performance mean, what is good and what is excellent performance mean? You need to mention compared to what expected level is your measure of discrimination is good?	other words, it measures how well the model can differentiate between instances that belong to different categories (for classification problems) or predict different values (for regression problems). A model with good discrimination power is able to assign higher probabilities or more confident predictions to the correct class or value, and lower probabilities to the incorrect ones. In classification tasks, where the model distinguishes between multiple classes (e.g., predicting whether a patient has a treatment failure or not).<br>Regarding performance:<br>**For prediction Models**<br>➢ **Good Performance:**<br>Accuracy: A model with accuracy (the proportion of correct predictions) around 70%-80% is generally considered to have good performance, depending on the complexity of the problem. In highly imbalanced datasets, though, accuracy can be misleading.<br>AUC (Area Under the ROC Curve): A model with an AUC score around 0.7 to 0.8 is considered good. This indicates that the

	model can effectively distinguish between classes.<br>Precision and Recall: If the dataset is imbalanced (e.g., rare events), a model with precision and recall values in the 70%-80% range can be considered good. Precision measures the correctness of positive predictions, and recall measures how many of the actual positives are correctly identified.<br>F1 Score: An F1 score (the harmonic mean of precision and recall) in the range of 0.7 to 0.8 is also considered good, particularly in imbalanced class scenarios.<br>➢ **Excellent Performance:**<br>Accuracy: An accuracy above 85%-90% is often seen as excellent, but the exact threshold will depend on the problem's difficulty and requirements.<br>AUC: An AUC close to 1.0, typically 0.9 or above, signifies excellent discrimination power. This means the model is nearly perfect at distinguishing between the positive and negative classes.<br>Precision and Recall: A precision and recall score near 90% or higher suggests that the

	model is making accurate and comprehensive predictions. A high recall ensures the model is identifying most of the positive cases, and high precision ensures it doesn't make too many false positive predictions.<br>F1 Score: A F1 score above 0.85 (or closer to 1.0) indicates excellent model performance in balancing precision and recall, which is particularly useful for imbalanced datasets.
The introduction lacks very important elements of your study that it deserves to show. Here is definition of treatment failure mean; you need to show at the beginning of your introduction, so that someone who reads your study will have a clear understanding.	Thank you dear reviewer for you suggestion. We admit the comment and correction has been made accordingly.
I haven't seen anything on the rate of treatment failure among patients with SCAP. You just over emphases rate of SCAP rather; you need to show more emphasis and show how much is the rate of occurrences of treatment failure, what studies say on the rate, this showed be well reviewed and included in the introduction.	Dear reviewer thank you for your comment. We have already mentioned it in line number 64.

In addition, there is no a single prediction study reviewed; do you mean there is no a prediction model conducted on treatment failure at all? Please you need to search studies, check what those studies lacks and need to show their limitation, and show write very good justification in the introduction section, only stating there no studies on the topic is not a good reason to convince your audience. I believe, you should have exclusively searched and included studies that benefit your study. Example here is a study which much closer to your interest here I dropped the link: https://pubmed.ncbi.nlm.nih.gov/36791115/. The study predicted poor outcomes of SCAP; treatment failure (prognostic outcome in the current study) was one of the composite outcomes in that study. Therefore, the authors in the current study should cite and state properly and can justify that the current study is unique from the previous study.	Dear reviewer, thank you for your comment. We mean no enough studies. The previous author measures the outcome variable of poor outcomes of severe community-acquired pneumonia" including treatment failure or antibiotic change or staying longer in the hospital or death occurred during follow-up days. Dear reviewer we have seen the article you mentioned. Your concern was also the concern the authors. We have discussed this issue in group. It was so controversial. Finally, we argued that comparing the current finding with the article you mentioned is not comparable. Because the previous study measures the outcome variable "poor outcome of SCAP"= treatment failure or antibiotic change or staying longer in the hospital or death occurred during follow-up days. But our outcome was only treatment failure of SCAP which was measured by persistence of features of severe pneumonia (cough and/or difficult breathing, with or without fever, fast breathing, or a lower chest wall in drawing where their chest moves in or retracts during inhalation) after initiation of antimicrobial

	therapy or a worsening clinical condition within 48–72 hours of the commencement of the antibiotics" The two measurement are quite different. So comparing the outcome with different measurement is difficult. To compare studies, we believe that outcome must be measured in similar way. Otherwise, our cooperation might be questionable again. Even if we consider this study is similar with the previous one, it is not sufficient. More studies are needed to investigate the problem. Additionally, the predictors to develop the prediction model in this study is not similar with the previous study. So models developed by different predictors is very important. That is why further study is considered. The time variation between the two studies may be another reason.
Line 89 to 98, is about the study design and setting, but there is no a single sentence about study period, although the title of this paragraph is study design, setting and period. Athough this study used a secondary analysis of the previous study; the authors should	Thank you so much dear reviewer. We tried to revise accordingly.

mention the theoretical time for this particular study.<br><br>They need to show exclusive time zero (time at which baseline measurement was taken) and the final time t1 at which the final scientific time for each individual patient included in the study, so that this study will have a correctly designed time frame.	Dear reviewer regarding the baseline measurement, we addressed in the methodology section such as in the operational definition section.
Sampling procedure, and data collection procedures, and data quality assurances methods from line 116 to 135 are all about sampling techniques and data collection methods used by the previous study, why you need to copy and put those things here, as you already used secondary data, you don’t have these techniques, as to me no need of having these issues here in your method section, just saying previous study is enough.	Thank you so much for your suggestion. We were tried to be more elaborative to avoid searching another article to see more about the sampling procedure and technique. Now we have made a revision based on your comment.
Line 151: “The prediction model was developed using a nomogram”, what by mean this, and rephrase such sentences in your work. You may sate that a nomogram tool was developed based on prognostic determinants found significant in the	Thank you so much. Amendment has made accordingly.

prediction model, or else in the way it is acceptable.	
Line 177: "the calibration/model fitness at an angle of 450 showed that there was no…", what by mean 450 angle? Please make correction???	Sorry dear reviewer, we mean 45^0.
Line 180, if your Youden index is 0.4389, what is "corresponding cut point 0.3585 probability mean? It is clear that Youden index is also predicted probability, please make clear for the audience here, between 0.4389, vs 0.3585	Thank you so much dear reviewer. We accept the comment and we revise the sentence accordingly to avoid possible misunderstanding.
Line: 191, "The risk prediction of SCAP treatment failure for the internally validated model using linear regression can be stated…."<br><br>Make correction to "linear regression" as linear predictors.	Thank you. Correction has made accordingly.
Discussion<br><br>Line: 241-245, you can't compare your model with such model, we can't do that while the models are totally different or incomparable; however, you may discuss your model, and state that model differently,	Dear reviewer it is very interesting comment. We feel that the article we tried to compare was slightly different in terms of predictors used in the model. But we believed that models developed in this study must be compared with the model developed in developed country and models developed

example you may state that your model is important in low income setting the determinants you used are easy to access and can be easily applied for decision making, however, that model you stated can also be important and can have a good accuracy in terms of stratifying patients based on risks also can't be applied in low income settings, or differently.	with different predictors. Instead comparing with special caution is important. That is why we tried to compare carefully like this "Even though the model was slightly different in terms of the included predictors and biomarkers like white blood cells, lactate, procalcitonin, cortisol, B-type natriuretic peptide, and C-reactive protein, the performance of our model was in line with a study conducted in a tertiary care university hospital in Portugal between December 2009 and March 2012 (AUC = 0.81, 95% CI: 0.74-0.91)". This study is similar with our study in terms of the outcome variable. The different is the predictors and the setting.
It is common to have potential limitations of studies, and even strengths if applicable, so where is yours?	Dear reviewer thank you so much for your comment. It is already mentioned in line number 367-371.
Conclusion<br>Should be well improved, refer my previous comment under the abstract section.	Dear reviewer, we have three objectives namely determine incidence of treatment failure, developing the model based on the prognostic factors and validating the model. Thus we tried to conclude based on these objectives.

Thank you so much for your very impressive scientific contribution.
