# Supplementary material for: Derivation and validation of a model to predict treatment failure among under five children with severe community acquired pneumonia who are admitted at Debre Tabor specialized comprehensive hospital
Source: PLoS One. 2025 Mar 26;20(3):e0320448. doi: 10.1371/journal.pone.0320448 (PMC11940812; doi:10.1371/journal.pone.0320448)
Supplement: Attachment — Submitted filename: #Derivation and validation treatment failure.docx [file pone.0320448.s005.docx]

#Derivation and validation of a model to predict treatment failure among under five children with severe community acquired pneumonia who are admitted at Debre Tabor specialized comprehensive hospital

Comments:

Abstract

The authors, described severe acquired pneumonia is challenging and only mentioned that treatment failure in SCAP is a severe condition, and stated that there is no study conducted to derivate and validate on treatment failure of SCAP. However, didn’t mention nothing what by mean treatment failure is about, it is good to state briefly for the readers of this article; even need to justify the need to conduct a prediction study; you need to tell the audience the importance of developing tool such as a nomogram or risk score in the background section.

Method:

Line 34 to 35; says “A multivariable analysis was conducted, and the model performance was evaluated using calibration and area under the curve”. You need to mention which multivariable analysis is that specifically that you used??

And need to mention that model performance was evaluated using ROC curve with its AUC and calibration curve; why AUC only mentioned there, it is incomplete in your case.

You need to mention, the descriptive statistics you used briefly in the method section, you overlooked, but findings were appeared in the result section.

The findings of this study showed that AUC for the original model is less than the validated model (line 41 to 42). How this scenario happened, general expectation is the metrics of the validated model is to be less, however, in your case the AUC is more optimized. I urge the authors to look back and rerun your validated model for accuracy. If still the result happened the same, you need to mention in what case it rarely happens and justify.

The conclusion part of your abstract is not well written, there is no conclusion made based on the determinants as one of your main objective to derivate a model, you are not only validating a model. In addition, you mentioned in your conclusion that your model had good discriminatory performance, is accuracy measures of a model only discrimination ability? What about calibration ability, even what about other measures of accuracy? Even what by mean good discrimination performance mean, what is good and what is excellent performance mean? You need to mention compared to what expected level is your measure of discrimination is good?

Introduction

The introduction lacks very important elements of your study that it deserves to show. Here is definition of treatment failure mean; you need to show at the beginning of your introduction, so that someone who reads your study will have a clear understanding.

I haven’t seen anything on the rate of treatment failure among patients with SCAP. You just over emphases rate of SCAP rather; you need to show more emphasis and show how much is the rate of occurrences of treatment failure, what studies say on the rate, this showed be well reviewed and included in the introduction.

In addition, there is no a single prediction study reviewed; do you mean there is no a prediction model conducted on treatment failure at all? Please you need to search studies, check what those studies lacks and need to show their limitation, and show write very good justification in the introduction section, only stating there no studies on the topic is not a good reason to convince your audience. I believe, you should have exclusively searched and included studies that benefit your study. Example here is a study which much closer to your interest here I dropped the link: <https://pubmed.ncbi.nlm.nih.gov/36791115/>. The study predicted poor outcomes of SCAP; treatment failure (prognostic outcome in the current study) was one of the composite outcomes in that study. Therefore, the authors in the current study should cite and state properly and can justify that the current study is unique from the previous study.

Methods

Line 89 to 98, is about the study design and setting, but there is no a single sentence about study period, although the title of this paragraph is study design, setting and period.

Athough this study used a secondary analysis of the previous study; the authors should mention the theoretical time for this particular study. They need to show exclusive time zero(time at which baseline measurement was taken) and the final time t1 at which the final scientific time for each individual patient included in the study, so that this study will have a correctly designed time frame.

Sampling procedure, and data collection procedures, and data quality assurances methods from line 116 to 135 are all about sampling techniques and data collection methods used by the previous study, why you need to copy and put those things here, as you already used secondary data, you don’t have these techniques, as to me no need of having these issues here in your method section, just saying previous study is enough.

Line 151: “The prediction model was developed using a nomogram”, what by mean this, and rephrase such sentences in your work. You may sate that a nomogram tool was developed based on prognostic determinants found significant in the prediction model, or else in the way it is acceptable.

**Results,**

Line 177: “the calibration/model fitness at an angle of 450 showed that there was no…”, what by mean 450 angle? Please make correction???

Line 180, if your Youden index is 0.4389, what is “corresponding cut point 0.3585 probability mean? It is clear that Youden index is also predicted probability, please make clear for the audience here, between 0.4389, vs 0.3585

Line: 191, “The risk prediction of SCAP treatment failure for the internally validated model using linear regression can be stated….”

Make correction to “linear regression” as linear predictors.

Line 196 to 2002, you developed a nomogram based on the 7 predictors, you need to show us how much is model accuracy for the nomogram model too! You only put model accuracy measures for the original model contain all predictors that were enter the multivariable analysis, however, your nomogram was developed based on only the 7, very important prognostic determinants found from the original prediction model. So, you also expected run a prediction model for the seven selected factors, then need to put all measures of accuracy such as ROC, with its AUC, calibration performances, and how much is the percentages of accuracy of nomogram model. This time you will be able to compare your nomogram model with the original model, then you will be showing the loss of accuracy in the nomogram model compared to the original one.

Where is sample of reading for your nomogram based on your predictors, you need to have sample of showing an individual risk of treatment failure(probability of developing failure) having attributes you included in the nomogram model.

Discussion

Line: 241-245, you can’t compare your model with such model, we can’t do that while the models are totally different or incomparable; however, you may discuss your model, and state that model differently, example you may state that your model is important in low income setting the determinants you used are easy to access and can be easily applied for decision making, however, that model you stated can also be important and can have a good accuracy in terms of stratifying patients based on risks also can’t be applied in low income settings, or differently.

It is common to have potential limitations of studies, and even strengths if applicable, so where is yours?

Conclusion

Should be well improved, refer my previous comment under the abstract section.
