# Supplementary material for: Derivation and validation of a model to predict treatment failure among under five children with severe community acquired pneumonia who are admitted at Debre Tabor specialized comprehensive hospital
Source: PLoS One. 2025 Mar 26;20(3):e0320448. doi: 10.1371/journal.pone.0320448 (PMC11940812; doi:10.1371/journal.pone.0320448)
Supplement: Attachment — Submitted filename: reviewer comments.docx [file pone.0320448.s003.docx]

**Comments to authors**

Generally, this is an extensive, novel, and well written research. With the following very minor comments the research work is publishable.

**Abstract:**

- Check the grammar
- Write the objective of the research separately
- Write the figures in to two digits

**Introduction:**

**In this section, the authors should address the following points**

- What is the clinical importance of the research?
- What is the gap of the previous research?

**Methods:**

- Use the appropriate punctuation for line 123.
- In line 159 please specify the figure.
- What is the importance of accuracy by chance value?
- Why you used a systematic random sampling?

**Results**:

- Please include the table within the main manuscript
- The model “P (SCAP treatment failure) = 1/e-(-2.67 +1.14 *HIV status yes +0.765* plural effusion (yes) +0.55 193 *anemia (yes) +0.647*measles (yes) +0.915*chest indrowing (yes) +1.07 *history of SCAP (yes) + 194 0.603*pulse rate (abnormal) (Table-3)” is not well written please revise it.
- Why you report decision curve analysis?

**Discussion:**

- If similar studies are available so far, please include additional comparisons.
- In line 228, what does it mean 450?
- What is the strength of the study?
- How to compare the AUC with corresponding previous AUC findings?
- What is your recommendations for researcher in the future?
